# Ubiquitination dynamics in human tumour viruses: Viral infection, oncogenesis and antiviral therapy

**DOI:** 10.1111/febs.70224

**Published:** 2025-08-17

**Authors:** Oscar Trejo‐Cerro, Martina Bergant Marušič, Justyna Broniarczyk

**Affiliations:** ^1^ Sir William Dunn School of Pathology University of Oxford UK; ^2^ Laboratory for Environmental and Life Sciences University of Nova Gorica Slovenia; ^3^ Department of Molecular Virology Adam Mickiewicz University Poznan Poland

**Keywords:** antiviral immune response, antiviral therapy, DUBs, E3 ligases, oncogenesis, oncoviruses, proteosome, ubiquitin, viral life cycle

## Abstract

The ubiquitin conjugation system is a critical regulator of cellular homeostasis and influences various cellular processes. Viruses, as obligate intracellular parasites, have evolved sophisticated strategies to utilise this system to enhance their survival, to either increase virus production or ensure the long‐term survival of the latently infected host. Viruses from almost all families, including RNA and DNA viruses, are challenged by ubiquitin‐mediated mechanisms at different stages of their life cycle and have evolved to exploit or bypass the host cell ubiquitination system for their own replication. In this review, we examine the diverse functions of the ubiquitin conjugation system during the different stages of viral infection, including viral entry, replication, gene expression, assembly and release. We discuss how human oncogenic viruses manipulate host ubiquitination pathways to maintain infection, evade immune responses and drive oncogenesis. Finally, we highlight new research aimed at uncovering the precise molecular interactions between oncoviruses and the host ubiquitination system, which will pave the way for the development of advanced therapeutic strategies to treat viral infections and cancer.

## Graphical abstract

The ubiquitin system is essential for cellular homeostasis and regulates many processes. Viruses, including oncogenic ones, exploit or evade this system to survive and replicate. This review explores how human tumour viruses manipulate the ubiquitination system to complete their life cycle, evade immunity and promote cancer. Understanding these interactions could reveal new therapeutic targets for treating viral infections and virus‐mediated cancer.

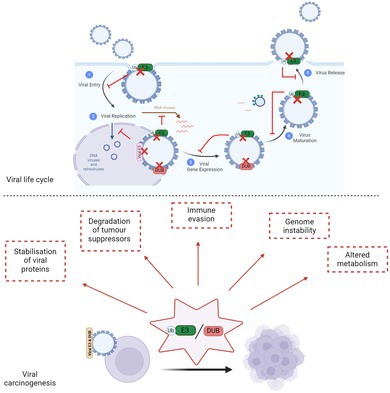

**Abbreviations**
AIDS: acquired immunodeficiency syndrome
ALT: adult T‐cell leukaemia/lymphoma
APC: anaphase‐promoting complex
DUB: deubiquitinating enzyme
EBNA: Epstein–Barr nuclear antigen
EBV: Epstein–Barr virus
ESCRT: endosomal sorting complexes required for transport
FBXO2: F‐box only protein 2
FDA: Food and Drug Administration
gB: glycoprotein B
HBV: hepatitis B virus
HBx: HBV X protein
HCC: hepatocellular carcinoma
HCV: hepatitis C virus
HECT: Homologous to the E6‐AP carboxyl terminus
HIV‐1: human immunodeficiency virus type 1
HLTF: helicase‐like transcription factor
HPV: human papillomavirus
HTLV‐1: human T‐cell lymphotropic virus type 1
HTS: high‐throughput screening
IARC: Agency for Research on Cancer
IFN: interferon
IKK: IκB kinase
IRF7: interferon regulatory factor 7
KSHV: Kaposi's sarcoma‐associated herpesvirus
LANA: latency‐associated nuclear antigen
LMP: latent membrane protein
LT: large T antigen
MCPyV: Merkel cell polyomavirus
MVBs: multivesicular bodies
NS5A: nonstructural protein 5A
NS5B: nonstructural protein 5B
PI: proteasome inhibitor
RBR: RING‐between‐RING
RING: really interesting new gene
ROS: reactive oxygen species
RTA: replication and transcription activator
sT: small T antigen
TLR: Toll‐like receptor
TPD: targeted protein degradation
Ub: ubiquitination
UBC: ubiquitin‐conjugating
UBE2S: ubiquitin‐conjugating enzyme E2S
UPS: ubiquitin–proteasome system
USP: ubiquitin‐specific protease

## An overview of the ubiquitin conjugation system

Ubiquitination (Ub) is a remarkably intricate enzymatic process in which an 8‐kDa ubiquitin molecule is bound to a specific lysine (Lys or K) site of the target protein [1]. This process involves a series of enzymatic reactions in which ubiquitin is transferred from the E1 ubiquitin‐activating enzyme to the E2 ubiquitin‐conjugating enzyme and finally to the E3 ubiquitin‐protein ligase, which then transfers the ubiquitin modification to the target protein. E1 enzymes catalyse the formation of a thioester bond between the C‐terminal carboxyl group of ubiquitin and a cysteine residue within the E1 enzyme itself. While E2 enzymes transfer the activated ubiquitin from E1 to E3 or directly to the substrate and play a key role in determining the type and length of the ubiquitin chain, E3 ligases provide substrate specificity and catalyse the final transfer of ubiquitin to the target protein, often interacting with both E2 and substrate [2]. The human proteome contains two ubiquitin‐specific E1 enzymes (UBA1 and UBA6), approximately 50 E2 enzymes, more than 600 E3 ligases and 100 deubiquitinating enzymes (DUBs) [3]. E1 enzymes are composed of multiple functional domains, such as the adenylation domain (AD), the catalytic cysteine domain (CCD) and the ubiquitin‐fold domain (UFD) [4]. The main characteristic of all E2s is a conserved ubiquitin‐conjugating (UBC) domain, which contains the E2‐active site and E3‐binding site. The E2 enzyme family is divided into four classes: Class I has only a UBC domain, Class II includes an N‐terminal extension, Class III has a C‐terminal extension, and Class IV contains both N‐ and C‐terminal extensions [5, 6]. A large proportion of ubiquitin conjugation system‐E3 ligases are categorised into four groups: Really Interesting New Gene (RING) Finger E3 ligases, Homologous to the E6‐AP Carboxyl Terminus (HECT), RING‐between‐RING (RBR), and U‐box E3 ligases. RING E3 ligases are the major group and contain an N‐terminal zinc‐binding RING domain that enables direct ubiquitin transfer from E2 to the substrate. The ubiquitination of HECT E3 ligases involves two steps: Ubiquitin is transferred from an E2 to the conserved C‐terminal HECT domain and then to the substrate. U‐box E3 ligases with a C‐terminal U‐box domain bind E2 and promote ubiquitin transfer. RBR E3 ligases feature two RING domains (RING1 and RING2) separated by a cysteine‐rich in‐between‐ring domain, facilitating a two‐step ubiquitin transfer via the catalytic RING2 domain [2].

A crucial role in the ubiquitin conjugation system is also played by DUBs, cysteine proteases that remove ubiquitin from target proteins and thus maintain a delicate balance in the final outcome of protein tagging [7, 8]. DUBs are classified into eight cysteine protease families with different structural features that determine their specific cleavage activity [9, 10].

Another important component of the ubiquitin system is the 26S proteasome, a large proteolytic complex responsible for the degradation of proteins that have been tagged with polyubiquitin chains for destruction [11, 12]. It comprises two main parts: the 20S core particle and two 19S regulatory caps [13, 14]. The 19S units recognise and bind to ubiquitinated proteins, remove the ubiquitin for recycling and unfold the target proteins before guiding them to the 20S core for degradation [15, 16].

**Fig. 1:**
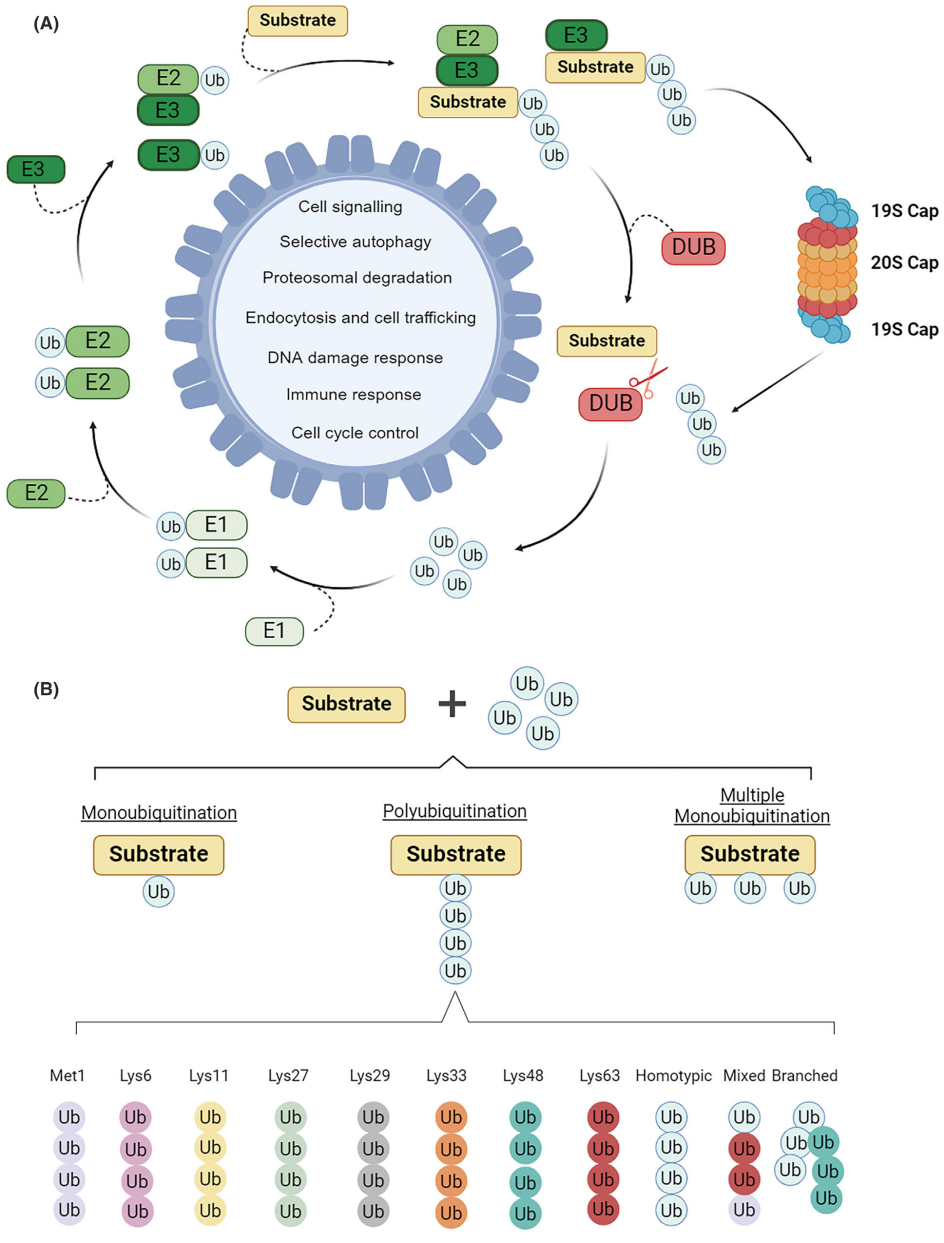
The ubiquitin‐proteasome system and its functions in the cell. (A) Schematic representation of the ubiquitination and deubiquitination reactions and examples of cellular functions that can be regulated by ubiquitination. This process involves a series of enzymatic reactions in which ubiquitin is transferred from the E1 ubiquitin‐activating enzyme to the E2 ubiquitin‐conjugating enzyme and finally to the E3 ubiquitin‐protein ligase, which then transfers the ubiquitin modification to the target protein. Deubiquitination is carried out by deubiquitinases (DUBs), which remove the ubiquitin modifications from the substrates (B) Schematic representation and examples of some of the ubiquitin modifications in the ‘ubiquitin code’. Figure created with BioRender.com

Ubiquitination, a reversible post‐translational modification, plays a pivotal role in regulating a diverse range of intracellular signalling networks. Beyond its involvement in proteasomal degradation, ubiquitination regulates numerous biological processes, including lysosomal degradation, intracellular trafficking, cell division, autophagy, cell signalling and immune response (Fig. 1A) [3, 7]. The versatility of the ubiquitin–proteasome system (UPS) in regulating a variety of processes arises from its unique ability to be conjugated as a monomer on one (monoubiquitination) or more (multiubiquitination) substrate lysines or as a polymer (polyubiquitination) (Fig. 1B). Polyubiquitination is created by linking one of the seven lysine residues of the ubiquitin (K6, K11, K27, K29, K33, K48 and K63) or the amino‐terminal methionine (M1) of ubiquitin to the carboxy‐terminal glycine residue of another ubiquitin molecule. Since Ub contains seven lysines, polyubiquitination can result in linear or branched chains with different topologies. The ‘ubiquitin code’ affects the function of these post‐translational modifications. For example, mono/multiubiquitination can regulate DNA repair, viral budding and gene expression, while K48 polyubiquitination generally leads to proteasomal degradation, and K63‐linked Ub chains play a role in signalling and endocytosis [17, 18, 19].

The consequences of dysregulated ubiquitin pathways are profound and far‐reaching and have an impact on the pathogenesis of many human disorders. These include cancer, metabolic syndromes, neurodegenerative diseases, autoimmunity, inflammatory disorders, muscular dystrophies and infectious diseases [20]. Understanding the complexities of ubiquitination is an essential step in developing effective strategies to combat these diseases.

This review addresses the complex role of the ubiquitin conjugation system in the mechanisms of tumour viruses' infection and oncogenesis. We explore how cancer‐associated viruses exploit the host ubiquitination machinery to evade immune responses and ensure viral persistence. In addition, the potential of targeting UPS components as a promising therapeutic strategy to inhibit oncogenic viral infections will be presented.

## Introduction to human tumour viruses

Based on the GLOBOCAN database of the International Agency for Research on Cancer (IARC), 13% of all new cancer cases in 2018 were attributable to infections. Apart from *Helicobacter pylori* and some parasitic infections, viruses are the main cause of infection‐related cancers, accounting for approximately 63% [21]. Seven oncogenic viruses classified by the IARC as well‐established Group 1 human carcinogenic agents are as follows: hepatitis B virus (HBV), hepatitis C virus (HCV), high‐risk types of human papillomavirus (HPV), Epstein–Barr virus (EBV), human herpesvirus type 8, also known as Kaposi's sarcoma‐associated herpesvirus (KSHV), human T‐cell lymphotropic virus type 1 (HTLV‐1) and human immunodeficiency virus, type‐1 (HIV‐1) [22]. Other viruses for which there is growing evidence of causality may be included as IARC Group 1 carcinogens in the future. Merkel cell polyomavirus (MCV or MCPyV) found in Merkel cell carcinoma [23] could be among the first candidates.

EBV, HPV, HTLV‐1, KSHV and MCPyV are direct carcinogens that promote cell transformation and cancer development. HBV and HCV are tumour viruses in which the oncogenic effect of the viral proteins is greatly enhanced by chronic inflammation in infected patients. Finally, HIV‐1 does not contain a known oncoprotein but causes cancer in patients with acquired immunodeficiency syndrome (AIDS) as a result of T‐cell depletion and the resulting immunosuppression [22]. Most tumour viruses have a DNA genome and are referred to as DNA tumour viruses, while HCV, HTLV‐1 and HIV‐1 belong to the group of RNA tumour viruses. General information on viruses known to be associated with cancer in humans is presented in Table 1.

**Table 1:** Human oncoviruses: genome features, tropism and their mechanisms of cancer development. (+), positive‐strand; ds, double‐stranded; EBNAs, Epstein–Barr nuclear antigens; EBV, Epstein–Barr virus; HBV, hepatitis B virus; HBZ, HTLV‐1 bZIP facto; HCV, hepatitis C virus; HIV‐1, human immunodeficiency virus type 1; HPV, human papillomavirus; HSCC, hypopharyngeal squamous cell carcinoma; HTLV‐1, human T‐cell lymphotropic virus type 1; KSHV, Kaposi's sarcoma‐associated herpesvirus; LANA, latency‐associated nuclear antigen; LMPs, latent membrane proteins; MCPyV, Merkel cell polyomavirus; ROS, reactive oxygen species; ss, single‐stranded.

Virus		Genome	Cell tropism	Associated cancers	Oncogenic proteins	Major cancerogenic mechanisms	References
DNA tumour viruses							
Papillomaviruses	HPV	Circular dsDNA (7.9 kb)	Mucosal and cutaneous epithelia	Cervical and other anogenital cancers, oropharyngeal cancers	High‐risk HPV E6 and E7 proteins	Inactivation of tumour suppressor proteins p53 and pRb, interference with the host cell signalling	[148, 149, 150]
Polyomaviruses	MCPyV	Circular dsDNA (5.4 kb)	Dermal fibroblast and other skin cell types	Merkel cell carcinoma (MCC)	Targe T (LT) and small T (sT) antigens	Inactivation of tumour suppressors, host genome instability	[151, 152]
Hepadnaviruses	HBV	Relaxed‐circular dsDNA (3.2 kb)	Hepatocytes	Hepatocellular carcinoma (HCC)	HBV X protein (HBx)	Direct HBV X‐related modulation of chromatin dynamics at specific gene loci Indirect inflammatory damage	[153, 154]
Gamma‐herpesviruses	EBV	Linear dsDNA (172 kb)	Epithelial and B cells	Nasopharyngeal and gastric carcinomas, Burkitt's lymphoma, Hodgkin's lymphoma, T‐cell lymphomas	EBNA, LMPs	Interference with the host gene expression Modulation of the host immune response	[155, 156]
	KSHV	Linear dsDNA (165 kb)	B cells, epithelial and endothelial cells, monocytes	Kaposi's sarcoma, Multicentric Castleman's disease, Primary effusion lymphoma	LANA	LANA‐related cell proliferation and suppression of apoptosis, Angio proliferative and inflammatory environment	[157, 158]
RNA tumour viruses							
Flaviviruses	HCV	ssRNA (+) (9.6 kb)	Hepatocytes	Hepatocellular carcinoma (HCC), non‐Hodgkin lymphoma	Core protein, NS3, NS5A, and NS5B	Dysregulation of cell signalling pathways Chronic inflammation; production of ROS, dysregulation of lipid metabolism	[159, 160]
Retroviruses	HTLV‐1	ssRNA (+) (9 kb)	CD4+ T cells and other lymphocytes	Adult T‐cell leukaemia (ATL)	Tax‐1, HBZ	Activation of NFκB, genomic instability Transcriptional activation of genes involved in T cell growth and transformation	[161, 162]
	HIV‐1	Dimeric ssRNA (+) (9.2 kb)	CD4+ T cells	Indirect contributing to the development of different cancers (Kaposi sarcoma, cervical cancer, non‐Hodgkin lymphoma)	No known oncogene	Depletion of CD4+ T cells and immunosuppression	[163]

Although human tumour viruses belong to different viral families and use different strategies to contribute to cancer development, they share many common features. One common characteristic that distinguishes them from other viruses is the tendency for long‐term persistent infections, in most cases with integration of the viral genome into the host DNA. Despite the viral aetiology of several cancers, infection with oncogenic viruses rarely leads to direct cancer development. According to epidemiological reports, carcinogenesis strongly depends on the viral load, the persistence of the infection and the contribution of host‐specific and environmental cofactors [22, 24]. Regardless of the exact mechanism underlying malignancy, viruses must utilise host machineries for replication and eventual cell transformation, with the UPS playing a crucial role in these processes.

For the purposes of this review, we will focus on tumour viruses with known oncogenes that cause cancer in humans. MCPyV is included due to its direct carcinogenesis, while HIV‐1 is not presented due to its strictly indirect role in cancer development and the absence of known oncogenes. For interested readers, the involvement of UPS in HIV‐1 infection has been discussed in detail in some recent reviews [25, 26].

## Ubiquitination in the entry, assembly and release of tumour viruses

Oncogenic viruses have developed various strategies to invade host cells, facilitating successful infection and potential oncogenesis. Although the entry, assembly and release mechanisms differ between viral families, all oncoviruses utilise the ubiquitin conjugation system to regulate these processes. Interestingly, ubiquitination can have both proviral and antiviral effects. It can promote viral entry, assembly and release by modifying host or viral proteins. Conversely, ubiquitination can also activate host defence mechanisms or alter viral proteins to disrupt fusion or assembly. For example, ubiquitination has been shown to be a host defence mechanism against EBV infection. EBV envelope glycoprotein B (gB) plays an essential role in the fusion mechanism required for the virus to enter B cells and epithelial cells. It was found that the E3 ligase F‐box only protein 2 (FBXO2) ubiquitinates and degrades N‐glycosylated gB via the ubiquitin–proteasome pathway, thereby suppressing EBV infectivity. On the other hand, depletion of FBXO2 stabilises gB and enhances its transport from the endoplasmic reticulum to the plasma membrane, which further promotes the membrane fusion and viral entry of the incoming viruses. FBXO2 is expressed in epithelial cells but absent in B cells, and its expression is upregulated in response to EBV infection [27, 28]. The EBV minor capsid protein BORF1 has also been shown to bind the E3 ligase TRIM5α, leading to BORF1 ubiquitination and its subsequent autophagic degradation. The resulting destabilisation of BORF1 can impair the transport of EBV capsid proteins into the nucleus, leading to defects in capsid assembly [28, 29].

Ubiquitination also plays a key role in promoting viral entry, trafficking and release. An interesting example is the association of the ubiquitin system with the host endosomal sorting complexes required for transport (ESCRT) during viral infection. ESCRT mediates crucial cellular processes such as endosomal membrane invagination, multivesicular body (MVB) formation, cell division, autophagy, membrane fission and plasma membrane repair. Many oncoviruses hijack the ESCRT machinery and ubiquitin components to enhance their entry into host cells and facilitate viral assembly and egress [30, 31, 32, 33, 34]. In KSHV infection, ubiquitination has been shown to mediate the internalisation of both KSHV and its receptor, integrin β1, with the E3 ligase c‐Cbl facilitating this process. KSHV hijacks c‐Cbl to monoubiquitinate the receptors and mark them for trafficking. This leads to the recruitment of ESCRT, which recognises the ubiquitinated proteins and transports them through various endosomal compartments, initiating macropinocytosis and infection [35].

Many enveloped oncoviruses utilise the ESCRT machinery to bud and release from infected cells. For example, the retrovirus HTLV‐1 requires the E3 ubiquitin ligase Nedd4 to ubiquitinate its major structural polyprotein, Gag, a key step for viral egress [36]. The E3 ligase Itch has also been shown to contribute to the release of HTLV‐1 [37]. HCV similarly exploits ESCRT by promoting K63‐linked polyubiquitination of its nonstructural protein NS2, which is critical for viral assembly and propagation [38]. Moreover, recent research has shown that HCV activates the ROS/JNK signalling pathway and the E3 ubiquitin ligase Itch, which promotes the release of HCV particles by polyubiquitination of VPS4A [39]. HBV follows a similar strategy and uses the MVB pathway in the late phase of infection [40]. Recent studies have shown that the E3 ubiquitin ligase Nedd4 plays a crucial role in this process by ubiquitinating the capsid/core protein HBc, facilitating its interaction with TSG101 and promoting ESCRT‐dependent viral release via MVBs [41].

**Fig. 2:**
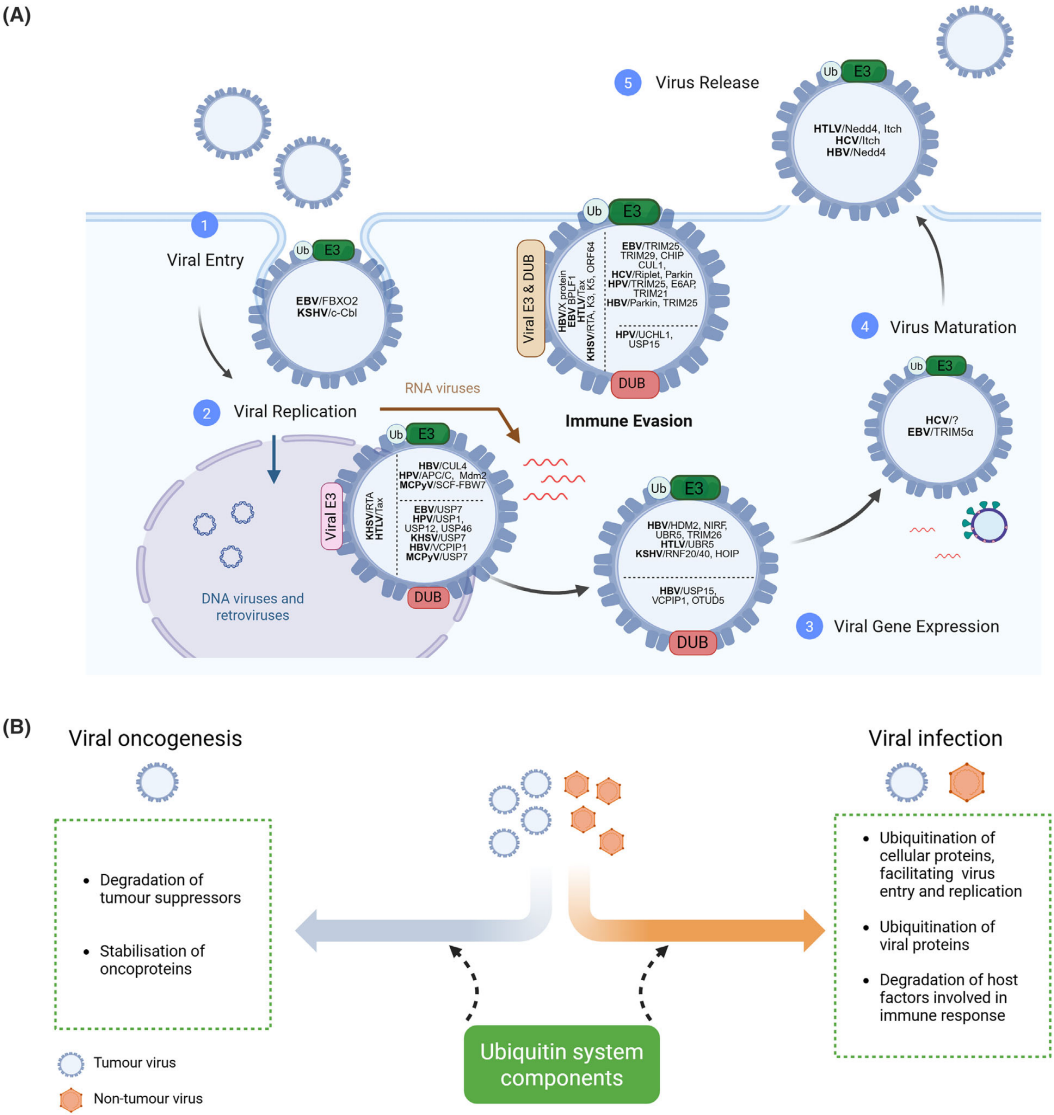
The role of the ubiquitin conjugation system in tumour virus infection and carcinogenesis. (A) Both host‐ and virus‐encoded E3 ligases and deubiquitinases (DUBs) play a crucial role in regulating key stages of the oncovirus life cycle, such as entry, replication, maturation, release, and immune evasion. At each stage of the viral life cycle, the main components of the ubiquitination system are shown in relation to a particular oncovirus. (B) The ubiquitin system plays a dual role during viral infection and induction of carcinogenesis. Figure created with BioRender.com

Although the role of ubiquitin in the immune evasion and the persistence of tumour viruses, as well as in virus‐induced carcinogenesis, is relatively well‐understood, its impact on tumour virus entry, intracellular trafficking, assembly and release is still largely unexplored. To date, only a limited number of components of the ubiquitin conjugative system, such as the E3 ligases c‐Cbl, Nedd4 or Itch, have been identified as regulators of these processes (Fig. 2A). A better understanding of these mechanisms could contribute to the development of new antiviral therapies that target viral invasion of host cells and thus reduce the risk of developing virus‐associated malignancies.

## From viral replication to carcinogenesis: Role of the ubiquitin system

One of the earliest evidence that viruses can modulate the host ubiquitin system to regulate the cell cycle for viral replication comes from studies on HPV, which demonstrated that HPV E6 drives the degradation of host p53 [42, 43]. Since then, it has become increasingly clear that viruses from most viral families exploit the host ubiquitination and deubiquitination machinery to facilitate their replication at different stages of their life cycle (Fig. 2A). Paradoxically, once the tumour forms due to infection by oncogenic viruses, viral replication is generally absent. However, persistent viral modulation of the ubiquitin system appears to be important for carcinogenesis and maintenance of the transformed phenotype (Fig. 2B).

### Ubiquitin‐mediated stabilisation of viral proteins

Regulating the expression and stability of viral proteins is essential for successful infection. In many cases, infected cells promote the degradation of viral proteins as a defence mechanism. For instance, the E3 ligase SPSB2 binds to the HCV protein NS5A via the C‐terminal SPRY domain, leading to its degradation and inhibiting viral replication [44], while the HBV core protein (HBc) is targeted by the E3 ubiquitin ligases NIRF and UBR5, affecting HBV gene expression [45, 46]. However, viruses can regulate the activity of ubiquitin ligases to evade ubiquitination‐mediated degradation. To counteract proteasomal HBc degradation, HBV upregulates the deubiquitinase OTUD5, which prevents HBc degradation by deubiquitinating the viral protein [47]. Although ubiquitination is primarily associated with protein degradation, it can also stabilise viral proteins. For example, TRIM26 interacts with HBc and prevents its ubiquitination and degradation [48], while the E3 ligase HDM2 increases the stability of the HBV X protein by NEDDylating it, and thereby preventing its degradation [49]. Interestingly, some viruses encode their own E3 ligases to regulate their viral expression. KSHV replication and transcription activator protein (RTA), a master regulator of the viral lytic cycle [50], possesses E3 ligase activity that facilitates its own ubiquitination and regulates RTA levels at different stages of infection [51, 52].

**Fig. 3:**
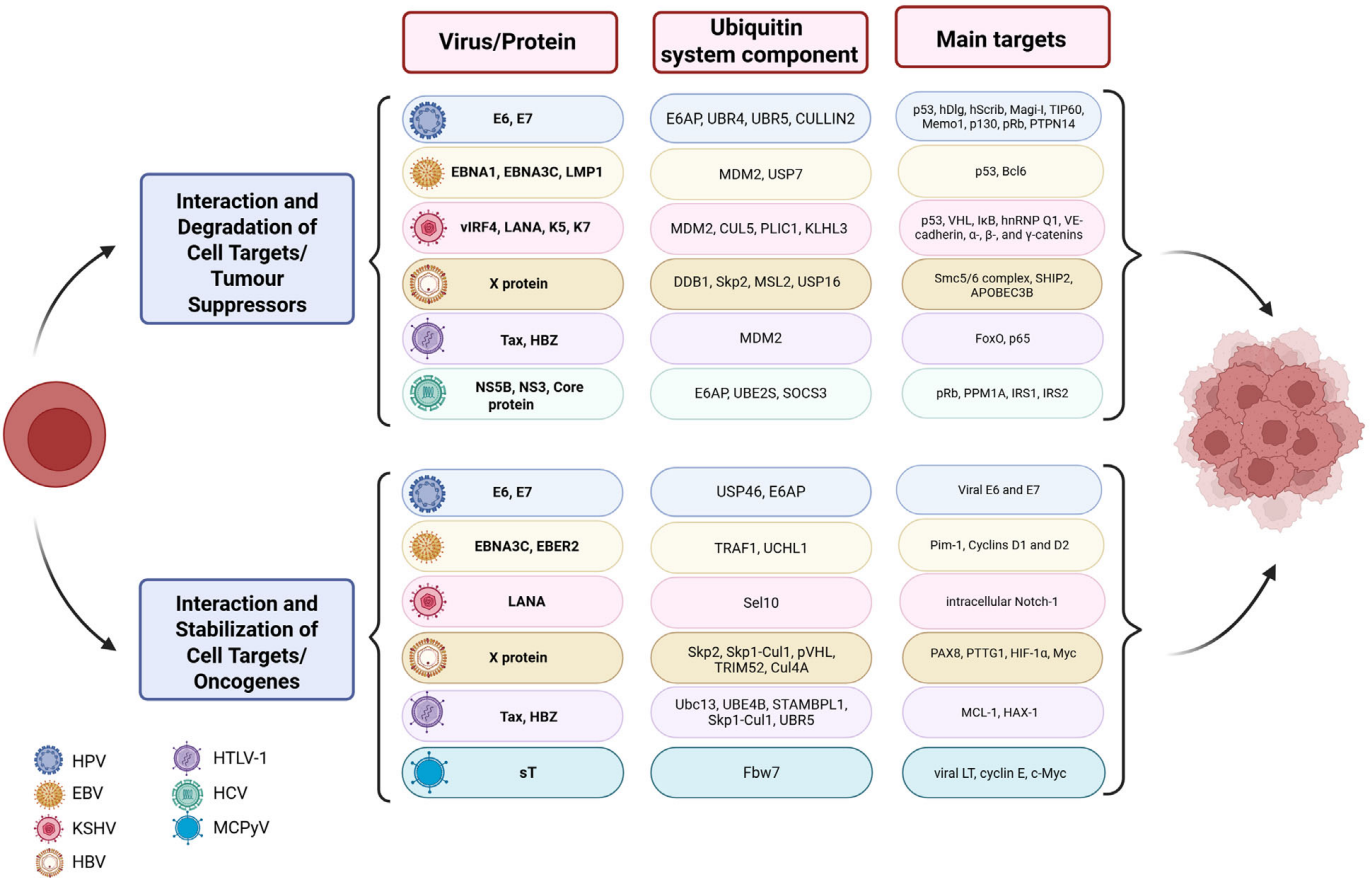
Ubiquitin conjugation system and virus‐associated oncogenesis. Human oncoviruses interact with the ubiquitin system, which in some cases contributes to cancer development. The indicated viral proteins modulate specific components of the ubiquitin system, often leading to either degradation or stabilisation of cellular targets, including the viral proteins themselves. Figure created with BioRender.com

The regulation of viral protein levels by the ubiquitin system is not only critical for viral infection but also plays a role in carcinogenesis (Fig. 3). The continued expression of viral oncoproteins, which in many cases is regulated by the ubiquitin system, can drive the progression of cancer. This is certainly the case in HPV‐induced carcinogenesis, where E6 and E7 harbour transforming potential and are key factors in the maintenance of the malignant phenotype [53]. The HPV oncoproteins E6 and E7 are stabilised by interaction with the E3 ligase E6AP, which enhances the proliferation of cervical cancer cell lines [54]. Another example is the HTLV protein HBZ, which is the only viral gene that is consistently expressed in infected patients and adult T‐cell leukaemia/lymphoma cell lines [55]. HBZ is ubiquitinated by UBR5, which modulates its stability. More importantly, UBR5 is essential for maintaining the proliferative phenotype of transformed T‐cell lines [55]. In hepatocellular carcinoma (HCC), high level of hepatitis virus X protein is frequently observed in HCC patients, and it has been associated with HCC progression [56]. Interestingly, the X protein can interact with the deubiquitinase USP15, which reduces X ubiquitination [57]. The protection of X protein from degradation by USP15 may represent an important mechanism for X protein stabilisation and the development of HBV‐related hepatocarcinoma [57].

### Role of the ubiquitin system in the regulation of tumour suppressors and oncogenes

The degradation of tumour suppressors and the stabilisation of host oncogenes are two hallmarks of the development of malignancy by tumour viruses. In the case of small DNA tumour viruses such as HPV, the disruption of the normal cell cycle is a prerequisite for a successful viral genome replication. This is mainly achieved by HPV E7‐mediated degradation of pRb via Cullin‐RING E3 ubiquitin ligase complexes [58]. However, the replicative environment of infected cells can activate apoptotic pathways. To counteract this, HPV E6 promotes ubiquitin‐mediated degradation of p53 via E6AP, which prevents p53‐mediated apoptosis [59]. EBV can also regulate the level of p53 through two complementary mechanisms: the Epstein–Barr nuclear antigen 3C (EBNA3C) interacts simultaneously with the nuclear‐localised E3 ubiquitin ligase Mdm2 and p53, which leads to ubiquitination and degradation of p53 [60]. Additionally, the EBV protein EBNA1 competes with p53 for binding to USP7, which prevents p53 deubiquitination [61]. Another interesting example is the KSHV protein LANA, which targets p53 via an unconventional intrinsic E3 ligase activity code for the viral protein [62]. LANA associates with the Elongin BC Complex and Cul5 through its SOCS‐Box–Like Motif, which increases the stability of LANA. Moreover, LANA–Elongin BC–Cul5 promotes ubiquitin‐proteosome degradation of the tumour suppressors VHL and p53, which creates a favourable environment for the progression of KSHV‐infected tumour cells [62]. During HCV infection, the viral RNA‐dependent RNA polymerase NS5B forms a complex with pRB, targeting it for degradation [63]. The ubiquitin ligase activity of E6AP is required for ubiquitination and degradation of pRB by NS5B, suggesting that oncoviruses may exploit similar ubiquitin components to degrade key tumour suppressors. This shared dependence on E6AP—in both HPV‐driven p53 degradation and HCV‐driven pRb degradation—emphasises the potential for targeting specific components of the ubiquitin system as a broader therapeutic strategy.

Destabilisation of p53, pRb and other tumour suppressors via the ubiquitin system appears to be a common pathway during viral oncogenesis. However, oncoviruses can also manipulate the ubiquitin system to activate oncogenes (Fig. 3). For example, in MCPyV‐induced oncogenesis, the sT antigen targets the ubiquitin ligase Fbw7 to inhibit proteasomal degradation of the viral LT and key cell cycle regulators such as cyclin E and Myc [64]. While the accumulation of these oncoproteins may contribute to carcinogenesis, the precise role of Fbw7 in Merkel cell carcinoma remains controversial [65]. Similarly, HBV enhances the stability of Myc by blocking its Skp2‐mediated ubiquitination, which contributes to oncogenesis [66]. EBV also exploits the ubiquitin system to stabilise oncogenic factors. The EBV protein EBNA3C inhibits ubiquitination and degradation of Pim‐1 and cyclins D1 and D2, thereby promoting cell proliferation [67, 68, 69]. Additionally, MCL1, a protein frequently overexpressed in cancer, is stabilised by HTLV HBZ, which disrupts the Skp1‐Cul1 complex and thus prevents the degradation of MCL1 [70].

### Ubiquitination as a driver of genome instability and metabolic reprogramming

The dysregulation of cell cycle regulators not only facilitates viral replication but also leads to DNA damage and genome instability, key hallmarks of cancer. For instance, HPV E2 modulates the APC/C ubiquitin ligase complex, affecting the stability of cell cycle regulators and host genome [71]. In the case of HTLV infection, an activation of the E3 ligase RNF8 by the viral Tax protein was observed. Activation of RNF8, a key regulator of DNA damage repair and cell division, has been associated with genomic instability in HTLV‐1‐transformed T cells [72]. Similarly, the MCPyV sT antigen can induce centrosome overduplication, aneuploidy and chromosome breaks by targeting E3 ubiquitin ligases [73], which may contribute to Merkel cell carcinoma development. Another interesting example is the ubiquitin‐conjugating enzyme E2S (UBE2S), which plays an important role in chromatin ubiquitination [74]. It has been reported that depletion of UBE2S increases HCV viral infectivity [75]. Moreover, the HCV protein NS5A interacts with UBE2S, impairing UBE2S‐mediated K‐11‐linkage modification in host chromatin and rendering HCV‐infected cells more sensitive to DNA‐damaging reagents [75]. The low levels of UBE2S detected in HCV‐infected cells suggest that this virus counteracts the antiviral activity of UBE2S and simultaneously contributes to HCV‐induced oncogenesis. Some oncoviruses not only target host ubiquitin enzymes but also encode their own ubiquitin components. The EBV protein BPLF1 is a lytic cycle protein with deubiquitinating activity that interferes with DNA damage repair and induces double‐strand breaks by deubiquitinating histone H2B, which blocks homologous recombination and increases genomic instability [76].

Another common mechanism of oncoviruses is to rewire metabolic signalling pathways through the ubiquitin system. During KSHV infection and in Kaposi's sarcoma lesions, a reduction in the levels of the RNA‐binding protein hnRNP Q1 was observed [77]. The KSHV protein vIRF1 recruits the E3 ubiquitin ligase Kelch‐like 3 (KLHL3) to degrade hnRNP Q1, leading to the induction of aerobic glycolysis [77]. This metabolic reprogramming is also known as the Warburg effect and represents a key process in carcinogenesis [78]. In HCC, HBV infection is associated with reduced levels of the SHIP2 protein, a regulator of insulin signalling [79]. The HBV protein X recruits the Skp2 E3 ligase complex to downregulate SHIP2, which promotes glucose uptake and cell migration in HCC cells [80]. Similarly, HCV infection leads to the degradation of the insulin receptor substrate proteins IRS1 and IRS2 via SOCS3‐mediated ubiquitination, which promotes insulin resistance and fibrotic progression [81].

All viruses utilise the cellular mechanisms of the host for their effective replication, and oncogenic viruses are no exception. However, oncoviruses appear to use components of the ubiquitin system more efficiently to degrade tumour suppressor genes, stabilise oncogenes, modulate cell cycle regulators and promote genomic instability, as well as alter metabolic signalling pathways, all of which contribute to cell transformation and cancer development.

## Ubiquitination in the immune evasion and persistence of tumour viruses

Although the degradation of tumour suppressors by oncoviruses represents one of the main factors in virus‐induced carcinogenesis, there is evidence that evasion of the host immune response also plays a fundamental role in this process. Interestingly, both mechanisms, tumour suppressor and immune signalling pathways, are associated with cell cycle arrest and induction of apoptosis in infected cells [82]. Inhibition of the antiviral immune response also enables sustained viral infection, which in many cases is crucial for the progression of malignancy. Importantly, viral manipulation of the ubiquitin system plays a key role in modulating immune responses regulation.

### Ubiquitin‐mediated degradation of immune components

Virus‐induced degradation of immune factors is one of the main mechanisms for immune evasion in persistent viral infections of many oncoviruses. The proinflammatory cytokine IL‐1β is degraded by HPV E6 via the ubiquitin ligase E6AP and p53 [83]. On the other hand, HPV E7 can recruit the E3 ligase TRIM21 to promote ubiquitination and degradation of the IFI16 inflammasome, revealing a novel mechanism of HPV immune evasion [84]. EBV promotes degradation of STING and RIG‐1 via the proteasome by interacting with the E3 ligases TRIM29 and CHIP, respectively [85, 86]. The binding of HCV core protein to STAT1 leads to the degradation of STAT1 via the proteasome [87], while HTLV‐1 can induce the expression of SOCS1, which inhibits Interferon‐β (IFN‐β) production by promoting ubiquitination and proteasomal degradation of IRF3 [88]. In the case of KSHV, the viral RTA protein acts as an E3 ligase that degrades IRF7, IRF3, MyD88, TRIF and HLA‐DRα via the ubiquitin–proteasome and blocks the Toll‐like receptor (TLR) signalling pathway [52, 89, 90, 91, 92].

### Inhibition of immune response activation via the ubiquitin system

Oncoviruses can also interfere with the immune response by preventing the ubiquitination and activation of immunomodulators. The HBV polymerase protein interacts with and disrupts the K63‐linked polyubiquitination of STING, inhibiting its activation and blocking the induction of IFN‐β [93]. Similarly, the HTLV protein Tax interacts with STING and reduces its K63‐linked ubiquitination, which in turn reduces IFN‐I production [94]. The polyubiquitination of RIG‐I is also required for its activation [95]. However, some oncoviruses target E3 ligases to prevent ubiquitination and activation of RIG‐I. This is the case with the HCV protease NS3‐4A, which targets the E3 ubiquitin ligase Riplet and reduces the polyubiquitination of RIG‐I [96]. The EBV protein BPLF1 forms a complex with 14–3‐3 and the TRIM25 ubiquitin ligase and promotes autoubiquitination of TRIM25, thereby preventing ubiquitination of RIG‐I and inhibiting the downstream type I IFN response [97]. Moreover, tumour viruses can also specifically target deubiquitinases to evade the immune response. For example, the cellular deubiquitinase UCHL1 is upregulated by HPV, which reduces the production of type I interferons, proinflammatory cytokines, and chemokines [98]. Interestingly, the HBV protein X can act as a deubiquitinase and prevent the ubiquitination and activation of cellular factors involved in the immune response, such as RIG‐I, IRF3, IRF7 and TRAF3 [99]. KSHV encodes ORF64, a tegument protein with deubiquitinase activity that reduces the ubiquitination of RIG‐I and attenuates its activity [100]. Similarly, the EBV protein BPLF1 can act as a functional deubiquitinase in infected cells, an activity that is critical for the attenuation of TLR signalling activation [101].

These are just a few examples of how oncoviruses can utilise the UPS to inhibit the host's immune response. However, they show that persistent infections, which are characteristic of oncoviruses, provide a favorable environment for long‐term expression of viral oncoproteins, prevention of apoptosis and consequently for cancer development.

## The ubiquitin conjugation system as a potential therapeutic target in oncovirus‐mediated pathologies

Modification of proteins with ubiquitin facilitates many steps of the viral life cycle, helps viruses evade the immune response and regulates virus‐associated carcinogenesis mechanisms, suggesting potential targets for antiviral therapies (Figs 2, 3). A deeper understanding of how this system interacts with cancer‐causing viruses could, therefore, lead to the discovery of new therapeutic strategies to block infections and prevent the development of virus‐induced cancers. Several components of the ubiquitin conjugation system (such as the proteasome, E1, E2, E3 and DUBs) have been investigated as potential therapeutic targets for oncogenic viruses.

The proteasome became the first successful therapeutic target within the ubiquitin–proteasome system and changed approaches to cancer treatment. Proteasome inhibitors (PIs) are a specialised class of drugs that interfere with the proteasome, preventing the degradation of key regulatory proteins that control cell division and programmed cell death, leading to an accumulation of proteins that can trigger cancer cell apoptosis. This mechanism has made these inhibitors particularly effective in the treatment of multiple cancers [102]. The natural compound lactacystin, derived from *Streptomyces* bacteria, was the first proteasome inhibitor discovered [103, 104, 105], while the peptide aldehyde MG132 was one of the earliest synthetically produced proteasome inhibitors [106, 107]. Bortezomib is the first and most successful of the PIs used in cancer therapy. It inhibits the NF‐kB signalling pathway, which is often associated with cancer progression, by reversibly targeting the 20S proteasome subunit and thus preventing the degradation of NF‐kB inhibitors [108, 109]. The drug has been approved by the US Food and Drug Administration (FDA) for the treatment of multiple myeloma and has also been used for the treatment of mantle cell lymphoma, non‐small‐cell lung cancer and pancreatic cancer [110, 111, 112, 113, 114, 115].

Although proteasome inhibitors were originally developed for cancer treatment, they have emerged as promising candidates for antiviral therapies because they can interfere with key processes that many oncoviruses rely on in host cells (Table 2). Several cancer‐associated viruses depend on the host proteasome at different stages of their life cycle, for example during viral entry (KSHV), replication (HBV), maintenance of viral protein stability (HBV, HPV and EBV) and evasion of immune recognition (EBV) [116, 117, 118, 119, 120, 121, 122, 123, 124]. Interestingly, some of the PIs also can reactivate tumour viruses (e.g. KHSV and EBV) from latency and initiate a lytic cycle [125]. However, the clinical application of proteasome inhibitors as antiviral agents is still in the early stages. Most ongoing clinical trials are focussed on virus‐induced cancers, where these inhibitors' dual antiviral and anticancer properties are being investigated. Unfortunately, the use of proteasome inhibitors is limited by their toxicity, nonspecific activity and the frequent occurrence of resistance. Attractive alternatives to overcome the limitations of proteasome inhibitors are offered by other components of the ubiquitin conjugation system and its upstream regulators, such as the E1–E2–E3 enzyme cascade or DUB enzymes, which have also been used as targets for cancer treatment [102, 126, 127, 128, 129, 130].

**Table 2:** List of inhibitors targeting components of the ubiquitin system that disrupt the life cycle of oncogenic viruses.

Name of compound	UPS target	Class	Oncogenic virus	Main mechanism	References
Bortezomib	Proteosome inhibitor	Peptide boronate	EBV	Activates viral oncoprotein degradation via autophagy‐lysosomal pathway	[124]
			HBV	Blocks viral replication	[117, 118]
			KHSV and EBV	Promotes the lytic cycle by activating JNK signalling and autophagy	[125]
Epoxomicin	Proteosome inhibitor	Peptide epoxyketone	KHSV	Reduces virus entry and intracellular trafficking	[116]
Lactacystin	Proteosome inhibitor	β‐lactone	EBV	Increase the ability of the immune system to recognise infected cells	[123]
MG132	Proteosome inhibitor	Peptide aldehyde	HPV, HBV and EBV	Regulates stability of viral oncoproteins and tumour suppressors	[119, 120, 121, 122, 124]
Nutlin‐3	MDM2 (E3 ligase)	Cis‐imidazoline analogue	EBV KHSV	Disrupts the interaction between MDM2 and p53, blocks p53 degradation and induces viral reactivation	[164, 165]
Bendamustine	E3 ligase linear ubiquitin assembly complex (LUBAC)	Benzimidazole	EBV	Reactivates the lytic cycle of the virus	[166]
Lenalidomide	E3 ligase cereblon (CRBN)	Isoindolones	EBV	Reactivates the lytic cycle of the virus	[167]
Pomalidomide	E3 ligase cereblon (CRBN)	Thalidomide analogue			
Thalidomide	E3 ligase cereblon (CRBN)	Phthalimides			
HBX 19818	USP7 (Deubiquitinase)	Aromatic amide	HPV	Disrupts the interaction between USP7 and HPV E7, leading to E7 degradation	[168]

Since the human genome encodes only two E1 enzymes, inhibition of either enzyme is likely to disrupt several different pathways, resulting in low specificity [4]. In contrast, E2 enzymes offer a much larger pool of potential targets for small‐molecule inhibitors. Numerous studies have shown that E2 ubiquitin‐conjugating enzymes are often dysregulated in cancer. Emerging evidence suggests that many E2s have an impact on DNA repair, cell cycle, oncogenic signalling and apoptosis during malignant transformation [131, 132, 133]. Despite the limited understanding of E2 function during oncovirus infection, it has been shown to play a crucial role in controlling viral replication (e.g. UBE2L3 and HBV), modulating host immune responses (e.g. UBC13 and HTLV‐1) and influencing the pathogenesis of virus‐induced cancers (e.g. UBE2S and HCV) [75, 134, 135]. Although no E2 inhibitors are currently approved by the FDA, some of them are in preclinical and early‐stage research, but mainly for the treatment of cancer and inflammation. Unfortunately, despite their highly conserved enzymatic core, these enzymes do not have defined catalytic pockets. Another major challenge in the development of E2‐targeting therapies is the risk of off‐target effects and side effects due to the broad range of substrates and functions regulated by E2 enzymes. Therefore, further research is needed to clarify the specific role of individual E2 enzymes in oncoviral pathogenesis and their contribution to virus‐induced cancers.

E3 ligases and DUBs are of particular interest due to their direct role in regulating the stability of target proteins. Many oncoviral proteins bind directly or indirectly to ubiquitin ligases (Table 3) and DUBs (Table 4) to facilitate viral intracellular trafficking and release (e.g. HTLV‐1 and HBV), to promote viral replication (e.g. EBV, HPV, KHSV, MCPyV), to alter critical cellular signalling pathways involved in the immune response (e.g. EBV, HPV) or to affect tumour suppressors such as p53 and pRb (EBV, HPV, HCV, KHSV), ultimately promoting malignancy. Therefore, targeting E3 ubiquitin ligases or DUB enzymes involved in tumour virus infections could be a promising therapeutic approach [9, 10, 136, 137, 138]. Due to their high substrate specificity, both ubiquitin ligases and deubiquitinating enzymes have a low risk of off‐target and side effects, which is related to the limited number of cellular events affected. Ideally, inhibition should block infection without affecting normal cells, but complex regulatory mechanisms can complicate this approach, and careful consideration of the target tissue or virus type is essential. For example, some enzymes, such as the ubiquitin ligase E6AP, can trigger HPV‐associated carcinogenesis through direct interaction with HPV E6 and increase the degradation of various tumour suppressors. In the case of HCV, E6AP targets the HCV core protein for proteasomal degradation, thereby reducing HCV infectivity, and E6AP inhibition could promote viral infection [59, 139]. Additionally, the complexity of ubiquitination and the lack of catalytic pockets in some E3 ubiquitin ligases, such as the RING and U‐box family, make them difficult to target compared to DUBs, which have a simpler mechanism and accessible catalytic pockets for small‐molecule binding. Despite these challenges, numerous small‐molecule inhibitors for human E3 ligases and DUBs have already been developed with varying degrees of success *in vitro*, in cell and animal models, and in clinical trials for cancer treatment. Interestingly, some of them targeting E3 ligases (MDM2, LUBAC, CRBN) or the USP7 deubiquitinase have also been shown to effectively disrupt the life cycles of oncogenic viruses such as EBV, KHSV and HPV.

**Table 3:** List of host‐encoded E3 ligases that interact with different proteins of tumour viruses and whose inhibition could disrupt the viral life cycle.

Name of component	Class of E3 ligase	Oncogenic virus/protein	Main mechanism	Function	References
CULLIN1/CULLIN2	RING	HPV/E7	Promotes the degradation of Memo1, p130 and pRb tumour suppressors	Oncogenesis	[58, 169]
CULLIN1	RING	EBV/BGLF2	Association and degradation of STAT	Immune evasion	[170]
CULLIN4/DDB1	RING	HBV/HBx	Degradation of Smc5/6 complex, which has an important role during DNA repair	Oncogenesis & Viral gene expression	[171, 172]
CHIP	U‐box ubiquitin	EBV/LMP1	Promotes RIG‐I degradation	Immune evasion	[86]
HDM2	RING	HBV/HBx	Increases HBx stability by promoting HBx NEDDylation	Oncogenesis	[49]
KLHL3	RING	KSHV/vIRF4	Degrades the RNA‐binding protein hnRNP Q1, inducing a metabolic reprogramming	Oncogenesis	[77]
E6AP	HECT	HPV/E6	Degrades proinflammatory cytokine IL‐1β	Immune evasion	[83]
			Degrades various tumour suppressors such as p53, Bak, hDlg, hScrib and Magi‐I	Oncogenesis	[59, 173, 174, 175, 176]
		HPV/E7	Increases the proliferation of cervical cancer‐derived cell lines	Oncogenesis	[54]
		HCV/NS5B	Targets pRb tumour suppressor for proteasomal degradation	Oncogenesis	[63, 177]
MDM2	RING	EBV/EBNA3C	Enhances p53 degradation, apoptosis inhibition	Oncogenesis	[60]
		KSHV/vIRF4			[178]
NEDD4	HECT	HTLV‐1/Gag	Ubiquitination of Gag	Enhancement of virus trafficking and release	[36]
		HBV/HBc	Ubiquitination of HBc		[41]
UBR5	HECT	HTLV‐1/HBZ	Modulates HBZ stability	Viral gene expression	[55]
TRAF6	RING	HTLV‐1/Tax	Ubiquitination of MCL‐1, which promotes the transformation of primary T cells	Oncogenesis	[179]
TRIM21	RING	HPV/E7	Promotes the ubiquitination and degradation of the IFI16 inflammasome	Immune evasion	[84]
TRIM25	RING	HPV/E6	Inhibits RIG‐I activation	Immune evasion	[180]
		EBV/BPLF1		Immune evasion	[97]
TRIM26	RING	HBV/HBc	Inhibits the degradation of HBc	Viral genome replication	[48]
UBR4	HECT	HPV/E7	Degrades PTPN14 tumour suppressor	Oncogenesis	[181]
UBR5	HECT	HTLV/HBZ	Modulates HBZ stability	Viral gene expression	[55]
		HPV/E6	Destabilises the histone acetyltransferase tumour suppressor TIP60	Oncogenesis	[182]
RIPLET	RING	HCV/NS3‐4A	Inhibits RGI‐I activation	Immune evasion	[96]
RNF8	RING	HTLV‐1/Tax	Affects DNA damage repair and cell division	Oncogenesis	[72]
SCF‐FBW7	F‐box complex Cullin RING	MCPyV/sT‐antigen	Prevents LT degradation	Viral Genome Replication	[64]
				Oncogenesis	
SCF‐SKP2	F‐box complex Cullin RING	EBV/EBNA‐3C	Cell cycle deregulation	Oncogenesis	[183]
		HBV/HBx	Downregulation of SHIP2, which promotes glucose uptake and cell migration in hepatocellular carcinoma cells	Oncogenesis	[80]
SMURF2	HECT	HCV/NS3	Induction of epithelial to mesenchymal transition by TGF‐β signalling	Oncogenesis	[184]

**Table 4:** List of host‐encoded deubiquitinases that interact with different proteins of tumour viruses and whose inhibition could disrupt the viral life cycle.

Name of component	Oncogenic virus/protein	Main mechanism	Functions	References
OTUD5	HBV/HBc	Prevents HBc degradation	Viral genome replication	[47]
USP1	HPV/E1	Promotes unidirectional replication of the HPV genome through the Fanconi anaemia DNA damage response pathway	Viral genome replication	[185, 186]
USP7	HPV/E7	Protect E7 from proteasomal degradation	Oncogenesis	[168]
	EBV/EBNA1	Regulation of p53 stability and inhibiting apoptosis	Oncogenesis	[61]
		Cleavage of monoubiquitin from histone H2B and thus activation of EBNA1‐related viral gene transcription	Viral genome replication	[187]
	KSHV/ LANA	Modulates the Replication of Virus Latent Episomal DNA	Viral genome replication	[188]
	KSHV/vIRF1	Inhibits p53‐mediated antiviral responses.	Immune evasion	[189]
	KSHV/vIRF1, vIRF3, and vIRF4	Unknown	Viral genome replication	[190]
	MCPyV/LT‐antigen	Unknown	Viral genome replication	[191]
USP12	HPV/E1	It is recruited by HPV E1 and UAF1 to the viral origin of DNA replication	Viral genome replication	[185, 186]
USP15	HPV/E6	Inhibits RIG‐I activation and the antiviral response	Immune evasion	[180]
	HBV/HBx	Increases HBx stability and its transactivation activity.	Unknown	[57]
USP46	HPV/E6	Activates proliferation of HPV‐transformed cancer cells	Oncogenesis	[192]
	HPV/E1	It is recruited by HPV E1 and UAF1 to the viral origin of DNA replication	Viral genome replication	[185, 186]

Notably, some complex oncoviruses encode viral ligases (e.g. KHSV and HTLV) and deubiquitinases (EBV, KHSV, HBV) that actively interfere with cellular ubiquitin‐dependent processes to suppress the host antiviral immune response and promote viral replication (Table 5). These viral enzymes have also become an important research focus in the development of antiviral therapies, as the inhibition of them could disrupt viral replication and prevent evasion of the immune response [140]. Indeed, inhibitors targeting the EBV BPLF protein, known for its deubiquitinase activity, have already demonstrated their efficacy and offer promising potential for new therapeutic strategies [141].

**Table 5:** List of virus‐encoded E3 ligases and deubiquitinases whose inhibition could specifically disrupt the viral life cycle and oncogenesis.

Oncogenic virus	Viral protein	UPS activity	Target protein	Main mechanism	References
EBV	BPLF1	Deubiquitinase	EBV ribonucleotide reductase	Reduction of the EBV ribonucleotide reductase activity	[193]
			kBa, TRAF6, NEMO	Suppression of TLR‐mediated activation of NF‐kB and immune evasion	[194]
			PCNA	Disruption of the cellular response to DNA damage	[195]
			TOP2	Stabilisation of topoisomerase‐II, enhancing cell survival and viral replication	[196]
HBV	HBx	Deubiquitinase	RIG‐I, IRF3, TRAF3, ikKi, MAVS, and STING	Inhibition of type I IFN‐mediated antiviral immune responses	[197, 198]
HTLV‐1	Tax	E3 ligase	NF‐κB	Activation of IKK‐NF‐κB signalling and evasion of the immune response	[199]
			HLTF	Production of defective virions and inhibition of the infectious replication cycle	[200]
KSHV	ORF64	Deubiquitinase	RIG‐I	Inhibition of RIG‐I‐mediated signalling	[100]
	K4, K6	E3 ligase	IFNG1, tetherin, CD1d and the MHC‐I	Immune evasion	[201, 202, 203, 204]
	RTA	E3 ligase	IRF‐7 and K‐RBP	Degradation of RTA repressors and lytic cycle progression	[51, 52]

These findings emphasise the urgent need to discover new inhibitors targeting the ubiquitin conjugation system to block oncogenic viral infections and prevent virus‐induced carcinogenesis. To achieve this, combined approaches using computational (*in silico*), structural biology, and biochemical methods are required. *In silico* approaches have become very popular as a first step in drug discovery and allow the screening of large virtual libraries of drug candidates. This approach has been made possible by the increasing availability of protein crystal structures and advanced docking and modelling programs that can predict binding between proteins and millions of compounds. Additionally, advanced bioinformatics tools utilising machine learning and artificial intelligence have further improved the speed and accuracy of these screenings and established *in silico* methods as a powerful tool in the search for new inhibitors [142, 143]. Another promising strategy to search for inhibitors targeting components of UPS is high‐throughput screening (HTS), which enables the simultaneous testing of thousands of compounds using fluorescence, chemiluminescence, or proximity assays. HTS, in conjunction with targeted protein degradation (TPD) technologies such as molecular glues and PROTACs, is a powerful tool for the discovery and validation of components of ubiquitin conjugative systems as potential drug targets. Molecular glues promote or induce the interaction between the E3 ubiquitin ligase and the target protein by modifying the surface of the ubiquitin ligase. In contrast, PROTACs induce target proteins to approach ubiquitin ligases by leading to ubiquitination and degradation of target proteins and providing a new way to screen, validate and optimise UPS inhibitors. Together, they open new avenues of targeted UPS modulation for therapeutic purposes [144, 145, 146, 147].

## Conclusions

The exploitation of the host ubiquitin system has emerged as a key strategy that enables tumour and other viruses to simultaneously overcome the host defences and exploit the cell machinery for their replication. The ubiquitin system, with its ability to modulate the stability, specificity and interaction dynamics of proteins, provides viruses with a powerful tool to fine‐tune the host cell proteome to their advantage. Understanding how viruses control the ubiquitin system to create their own ubiquitin code will provide crucial insights into which host factors are beneficial or detrimental for infection and, consequently, cancer development in tumour viruses. While much research has focused on the role of ubiquitin in immune response and oncogenesis, relatively little is known about its function in modulating tumour virus cell entry, intracellular trafficking, assembly and release. Although these processes may not be unique to oncogenic viruses, they represent attractive targets for the development of broader antiviral and preventive measures.

Future research should aim to uncover the full spectrum of UPS‐interacting factors involved in oncovirus pathogenesis. While several key E3 ligases and deubiquitinases involved in viral infection and oncogenesis have been identified, many remain unexplored. Advanced proteomics and genetic screening approaches may reveal new viral and host ubiquitin components that are critical for infection and transformation. Importantly, many UPS components are enzymes, indicating that it is not sufficient to monitor protein or mRNA expression levels, as these are not necessarily related to enzyme activity. This highlights the need for activity‐based probes, which can provide real‐time insights into the dynamic regulation of ubiquitin‐related enzymes during viral infection and tumour progression.

Targeting the UPS in the process of virus‐induced cell transformation and cancer requires a highly specific approach. Given the central role of the UPS in normal cellular processes, general inhibitors of E1, E2, or the proteasome carry a high risk of nonspecific responses, side effects and drug resistance. E3 ligases and DUBs, with their high specificity, seem to be much better suited, but selecting the most promising E3 and DUBs targets for a particular viral infection and associated cancer is not an easy task. However, a critical challenge remains: Should we prioritise targeting enzymes that regulate viral oncoproteins to achieve specificity, or focus on those that modulate cellular tumour suppressors and oncogenes to broaden the therapeutic impact?

New technologies offer valuable tools to increase the efficiency and specificity of small‐molecule inhibitors, which are currently the focus of interest. Targeting the UPS pathways in cancers with the multi‐step approach could represent a new way to specifically exploit the participation of UPS in virus‐related carcinogenesis with far fewer side effects. This, however, requires a thorough understanding of both the general and virus‐specific host‐pathogen interactions in which the UPS pathways are involved. Such insights are essential for the development of innovative antiviral therapies ensuring sufficient specificity to prevent deleterious disruption of normal ubiquitin‐regulated protein dynamics in the host. The interaction of viruses with the ubiquitin system is, therefore, both an effective tool for the study of complex cellular processes and a powerful therapeutic option for the treatment of tumour virus‐related pathologies.

## Conflict of interest

The authors declare no conflict of interest.

## Author contributions

OTC, MBM and JB contributed to the conceptualisation, writing, review and editing of this work. All authors have read and agreed to the published version of the manuscript.
